# Emergency Department of a Rural Hospital in Ecuador

**DOI:** 10.5811/westjem.2015.11.27936

**Published:** 2016-01-12

**Authors:** Tara Johnson, David Gaus, Diego Herrera

**Affiliations:** * Maricopa Medical Center, Department of Emergency Medicine, Phoenix, Arizona; † University of Wisconsin School of Medicine and Public Health, Department of Family Medicine, Madison, Wisconsin; ‡ Central University of Ecuador, Catholic University of Ecuador, Department of Family Medicine, Santo Domingo, Ecuador

## Abstract

**Introduction:**

There is a paucity of data studying patients and complaints presenting to emergency departments (EDs) in low- and middle-income countries. The town of Pedro Vicente Maldonado (PVM) is located in the northwestern highlands of Ecuador. Hospital PVM (HPVM) is a rural teaching hospital providing family medicine residency training. These physicians provide around-the-clock acute medical care in HPVM’s ED. This study provides a first look at a functioning ED in rural Latin America by reviewing one year of ED visits to HPVM.

**Methods:**

All ED visits between April 14, 2013, and April 13, 2014, were included and analyzed, totaling 1,239 patient visits. Data were collected from their electronic medical record and exported into a de-identified Excel® database where it was sorted and categorized. Variables included age, gender, mode of arrival, insurance type, month and day of the week of the service, chief complaint, laboratory and imaging requests, and disposition. We performed descriptive statistics, and where possible, comparisons using Student’s T or chi-square, as appropriate.

**Results:**

Of the 1239 total ED visits, 48% were males and 52% females; 93% of the visits were ambulatory, and 7% came by ambulance. Sixty-three percent of the patients had social security insurance. The top three chief complaints were abdominal pain (25.5%), fever (15.1%) and trauma (10.8%). Healthcare providers requested labs on 71.3% of patients and imaging on 43.2%. The most frequently requested imaging studies were chest radiograph (14.9%), upper extremity radiograph (9.4%), and electrocardiogram (9.0%). There was no seasonal or day-of-week variability to number of ED patients. The chief complaint of human or animal bite made it more likely the patient would be admitted, and the chief complaint of traumatic injury made it more likely the patient would be transferred.

**Conclusion:**

Analysis of patients presenting to a rural ED in Ecuador contributes to the global study of acute care in the developing world and also provides a self-analysis identifying disease patterns of the area, training topics for residents, areas for introducing protocols, and information to help planning for rural EDs in low- and middle-income countries.

## INTRODUCTION

The study of global emergency medicine (EM) systems is important, and further system-development research is necessary.^1^,^2^ Lower income countries have restricted resources including limitations in infrastructure, technology, supplies, manpower, and access to further education. The burden of disease can vary between locations, as can the “cultural, political, and social aspects of delivery of care.”^3^,^4^ A deeper understanding of acute care delivery systems and their patients is essential to providing better acute care. However, minimal data exist from emergency departments (EDs) in Latin America, and there is no information regarding acute care in Ecuador. The information available from Latin America focuses on urban and university hospitals only, and to our knowledge there is no data on acute care in a rural setting.^5^–^16^

Current literature calls for the need to characterize acute care systems by nation or region and then analyze broader regional EM trends, while also calling for more research in rural areas.^17^–^19^ There is also a call to focus on patient’s chief complaint for seeking care, not the final diagnosis, as the best way to understand global acute care settings and compare between settings.^1^,^19^

The country of Ecuador is located in northwestern South America, lies on the Equator, and is bordered by Colombia, Peru, and the Pacific Ocean. The country has different geographical regions including the coast, tropics, highlands, and the Amazon. In 2013, the population was nearly 16 million. The under-five mortality rate was 23 per 1000, compared to the regional average of 15 and the global average of 48. There were 17 physicians per 10,000 people compared to the regional average of 21.^20^ In Ecuador, 32% of the population is rural, and 42% of rural people live below the national poverty line. Twelve percent of the population lives at <$2/day, and 16% of the population is undernourished. Nearly 25% of rural Ecuadorians do not have access to an improved water source.^21^

Pedro Vicente Maldonado (PVM) is a small rural town located on the western slopes of the Andes Mountains at an altitude of 600m in northwest Ecuador, and is the catchment area for around 70,000 people living in both tropical and subtropical regions.^22^ Hospital PVM is a small, 15-bed hospital that serves this area and provides secondary level healthcare services such as inpatient medical, surgical, and obstetric-gynecologic services, some intermediate intensive care unit care, full-spectrum primary care clinical services, specialist outpatient care and day procedures, and includes a functioning emergency department that is open 24 hours a day, seven days a week.^23^,^24^

In Ecuador only a few large urban centers have dedicated EM specialists, and most EDs are staffed by physicians with varied levels of training, most without formal EM education. Hospital Pedro Vicente Maldonado (HPVM) serves as one of two main rural teaching hospitals for providing a formal family medicine residency-training curriculum, and these physicians provide 24-hour acute medical care in HPVM’s ED. This unique training program allows for improved experience, skills training, and supervision beyond the typical six-year combined medical school and rural internship undertaken by most generalists in the country.^23^–^26^

This study is the first descriptive analysis of a functioning ED in rural Latin America. While contributing to the global study of acute care medicine in the developing world, this study also provides an analysis of the HPVM ED that can be used to identify disease patterns of the area; define training topics for residents; identify areas for treatment guidelines and protocols; inform decisions regarding trauma education, triage strategies, and need for specialty care and more advanced technological resources while performing future ED planning; and inform local and national public health policy.

## METHODS

All ED visits to HPVM over a one-year period between April 14, 2013, and April 13, 2014, were included and analyzed, totaling 1,239 patient visits. Information from each patient’s visit was entered into an electronic medical record (EMR) system created by the hospital per usual ED practice. We the obtained information for purposes of this study from the EMR and abstracted the data into a de-identified and password-protected Excel® (2011) database where it was sorted and categorized.

**Figure 1:**
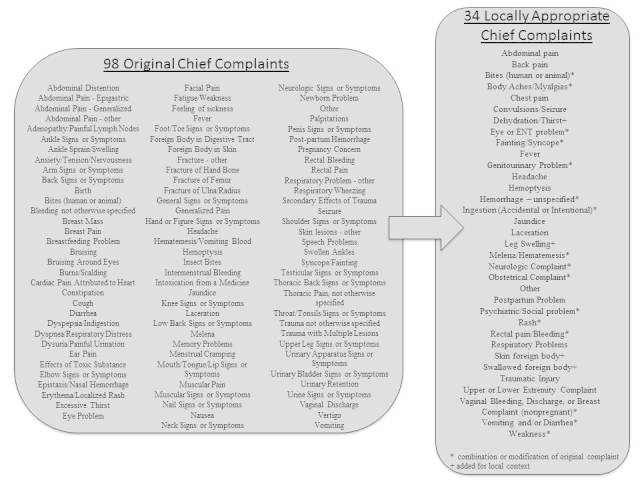
The development of categorization of locally appropriate chief complaints for Hospital Pedro Vicente Maldonado.

Information obtained included age, date of service, gender, mode of arrival, insurance type (government-provided social security insurance or not), chief complaint, laboratory and imaging requests, and disposition. To keep information de-identifiable, we converted date of service into month and day-of-the-week of service. For the analysis of chief complaint, initially the 1,239 patient visits were associated with 98 different chief complaints. In an effort to categorize into useful groupings, we used a starting list of 57 chief complaints for categorization based on previous efforts by Aronsky et al in 2001.^27^ We then modified these data into a more locally appropriate list of 34 complaints (Figure 1) which were then used in the analysis. Imaging requests were also further categorized from a free-text request into groupings according to imaging type and area of study. We performed descriptive statistics for all variables. Where possible, comparisons were performed using Student’s T or chi-square, as appropriate. The study was determined to be exempt by the institutional review board of Maricopa Integrated Health System, and was reviewed and permitted by the Andean Health Advisory Board.

## RESULTS

**Figure 2:**
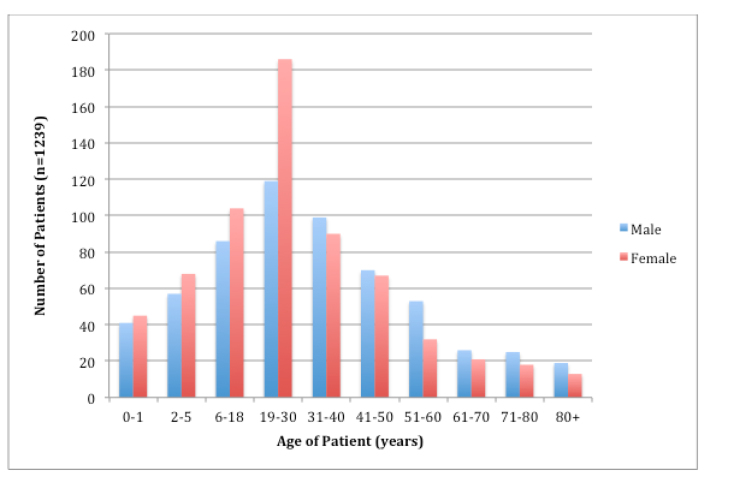
Age distribution of patients, by gender.

Between April 14, 2013, and April 13, 2014, there were a total of 1,239 ED visits for both adults and children, 48% male and 52% female; 24.6% of the patients were between age 19–30 years, and 15.3% were between 6–18yrs and 31–40 years. Nearly 7% were <1 year old (Figure 2). The average age of patients with a traumatic complaint was 31 years, and 67.9% of trauma patients were male and 32.1% were female. The average age of patients with an obstetric complaint was 25.5 years.

### Mode of Arrival

Of these patient visits, 93% were ambulatory, and 7% came by ambulance. Those who came by ambulance were more likely to have a chief complaint of traumatic injury (49.4% vs. 8%, p<0.0001). Those who were ambulatory arrivals were more likely to have a chief complaint of abdominal pain (26.8% vs. 7.2%, p=0.0001), back pain (5.7% vs. 0%, p=0.049), and fever (16.1 vs. 1.2%, p=0.0005). Those who came by ambulance were more likely to be admitted (33.7% vs. 19.7%, p=0.0039) or transferred (34.9% vs. 10.8%, p<0.0001) when compared with ambulatory patients. Ambulatory patients were more likely to be discharged home (39.9% vs. 21.7%, p=0.0016) or sent to clinic (29.2% vs. 8.4%, p<0.0001).

### Chief Complaint

The classification of chief complaints into their final categories is shown in Figure 1. The top three chief complaints for all-comers were abdominal pain (25.5%), fever (15.1%) and trauma (10.8%). Chief complaints were also stratified by children 0–5 years, adolescents 6–18 years, adults 19–59 years, and elderly 60+ years (Tables 1–4).

### Insurance Status

Patient insurance type was recorded as farmer, general, retired, or “other.” Farmer, general, and retired insurance types are all government-provided social security insurance (hereon grouped as “social security insurance”). Farmer’s social security covers those who live in rural areas and can demonstrate that they work the land for a living and pay a very nominal monthly fee to the Ecuadorian Social Security Institute. General social security covers patients whose employers pay social security taxes. Retired social security affiliates are those who were previously in the general category but are now retired based on age. “Other” insurance is a catchment group that includes Obligatory Transit Accident Insurance (SOAT), a national plan covering medical care associated with motor vehicle accidents, police insurance, and assorted private insurance (the latter being infrequent). Unfortunately further delineation of this group is unavailable, although SOAT insurance is suspected to cover much of this.

Of those who arrived by ambulance 65.1% had “other” insurance, compared with 35.1% of those who were ambulatory (p<0.0001). Of all total complaints, patients with social security insurance were more likely to have the chief complaint of abdominal pain (28.0% vs 21.3%, p=0.011), fever (17.3% vs 11.3%, p=0.006), headache (1.9% vs 0.2%, p=0.02), and rash (0.6% vs 0.2%, p=0.033), compared with those with “other” insurance. Patients with “other” insurance were more likely to have the chief complaint of human or animal bite (2.4% vs 0.6%, p=0.013), ingestion (accidental or intentional) (1.5% vs 0.3%, p=0.042), traumatic injury (17.8% vs 6.7%, p<0.0001), and foot/leg pain (4.3% vs 1.7%, p=0.010), compared with those with social security insurance.

### Labs and Imaging

Of all patients presenting to ED, labs alone were requested on 693 (55.9%) patients. Imaging alone was requested on 344 (27.8%) patients. Both labs and imaging were requested on 190 (15.4%) patients. Eleven (0.9%) patients had neither labs nor imaging requested. Patients were more likely to have labs alone requested if they had chief complaint of fever, imaging alone requested if they had chief complaint of abdominal pain, laceration, traumatic injury, foot/leg pain, and both labs and imaging requested if they had chief complaint of respiratory problem. The most frequently requested imaging studies were chest radiograph (14.9%), radiograph of an upper extremity (9.4%), and electrocardiogram (9.0%). Other imaging available but used less frequently were radiographs of different body areas; abdominal, renal, and pelvic/genital ultrasounds; and fetal heart monitoring/non-stress tests. Of note, although a computerized tomography of the head or abdomen and pelvis may have been requested, these are not available at HPVM and patient either was transferred or did not receive this imaging.

Of all patients with a traumatic complaint, 92.5% had imaging requested but only 11.9% had labs requested. Of all patients with an obstetric complaint, 84.5% had imaging requested and 37.9% had labs requested. Of all pediatric patients <18 years, 32.0% had imaging requested and 75.5% had labs requested.

### Day of Week and Seasonal Variability

The rainy season in the area of HPVM is December through May, but there was no change in number of ED visits during this time when compared with the rest of the year. There was also no statistical difference in number of visits by day of week. This is also true in a subgroup analysis of pediatric patients and in patients with obstetrical and trauma complaints.

### Disposition

Of all patients presenting to the ED, 484 (39.0%) were discharged home, 256 (20.7%) were admitted to the hospital, 345 (27.8%) were sent to outpatient clinic, and 154 (12.4%) were transferred. Patients were more likely to be admitted if they had a chief complaint of human or animal bites and more likely to be transferred if they had a chief complaint of traumatic injury. Patients were less likely to be transferred if they had a chief complaint of back pain or fever.

Of those discharged to home, insurance type was not statistically different. However, of those admitted, 23.7% vs 15.4% had social security insurance (p=0.0007), and transferred patients were more likely to have “other” insurance (17.8% vs 9.2%, p<0.0001). Patient’s disposition from the ED was not statistically determined by gender, day of week or month of year presentation, or requests for imaging and/or labs.

## LIMITATIONS

Our data are limited in that they only include one year of data from one institution, and these data may or may not be generalizable to a larger setting within rural Ecuador, Latin America, or the greater developing world. Also, in many ways, having a “final diagnosis” or “ED impression” would be interesting and helpful when performing analysis on these patients, as this can be somewhat more tangible and familiar when comparing results with data and experiences from the developed world. Although the physicians seeing these patients listed a differential diagnosis, these lists of 4+ possibilities could not be analyzed in a useful way. Furthermore, previously published data recommended analysis based on chief complaint only and not diagnosis.^19^

Additionally, there is a lack of consensus on how to classify variables used in analyses of developing world ED systems. There is no formal universal listing of chief complaints or standard list of imaging studies that can be compared between studies,^17^ so classification for this study was performed starting from available lists from the developed world and was made to be locally appropriate. We did attempt to make an inclusive list that can be replicated in further studies, but it may not be appropriate for all areas. Additionally, analysis was limited to the available variables recorded in HPVM’s EMR system. A guideline for global ED data keeping and a guideline for variable analysis would be beneficial for both initiating data collection systems and for performing future comparative studies.

## DISCUSSION

This is the first study looking at a rural ED in Latin America. There is therefore little available information for comparison between other sites. This study underscores the importance of EMRs, as their use in this hospital allowed a comprehensive view of an entire year’s worth of patients. As HPVM accepts all patients regardless of age, gender, race, or financial status, all ED patients were included in the study. Unlike many studies having high rates of exclusions secondary to poor documentation, illegible handwriting, or research time burden associated with review of paper charting, electronic charting for these patients was relatively efficient and nearly always complete, allowing nearly full capture of the spectrum of ED care during this time period.

This study highlights the large number of patients between age 6–40, but still underscores the need for ability to treat patients of all ages, with 7% of patients seen being <1 year old and 6% >71 years old. Similar to data from the developing world, this study shows the high number of traumatic complaints from young male patients. It also shows the high number of obstetric complaints and the need for full-spectrum OB care in this ED – not just through the first 20 weeks of pregnancy like typical EDs in the United States. Given the variety of chief complaints and high frequency of abdominal pain complaints, improving point-of-care ultrasound skills of treating physicians may increase speed and accuracy of diagnosis.

This study shows the large discrepancy between number of ambulatory and ambulance visits. Further studies in rural Ecuador regarding ambulance access, reliability, and costs would be helpful to help better understand pre-hospital transport systems and acute care in the field. There were 1,239 visits to the ED during the one-year period. Although this seems quite small when compared with a typical rural ED in the U.S, no data are available for comparison in this setting. Additionally, although time of visit was not available for analysis, anecdotally a majority of patients came to the ED in the evening and overnight hours. Further analysis of patient decisions to seek care in the ED would be helpful for catering to patient acute care needs.

Insurance status did not overall seem to affect care received in the ED. Further studies of patient’s knowledge of health insurance and out-of-pocket costs will help ongoing system improvement and ED planning.

Finally this study highlights the large number of patients who are evaluated in the ED and then sent directly to clinic. An analysis of these patients may provide insight into the scope of rural outpatient care and help triage techniques for the management of chronic problems in an acute care setting. It also begins to assess the spectrum of services of a rural secondary hospital in subtropical Ecuador while also defining its limits and acknowledging circumstances requiring transfer to a higher level of care. A more detailed analysis of transferred patients would be beneficial to help determine areas for improvement of medical education curriculum and provider skill sets, define appropriate areas for allocation of limited resources, and set up transfer protocols.

## CONCLUSION

This is a descriptive study analyzing an acute care system in rural Ecuador, and it provides valuable information to others attempting to set up similar rural EDs. To be clear, however, this is not a needs assessment and or a proposal of ED resident curriculum at this point. Combining an understanding of the dynamics of health, public health, and politics, along with trained clinicians, appropriate infrastructure and staff, and the help of modern technology and resources, advances can continue to be made in rural acute care in Latin America.

## Figures and Tables

**Table 1:** Chief complaint by age, 0–5yrs.

Complaint	Total #	% of total
Fever	89	42.2%
Vomiting and/or diarrhea	38	18.0%
Respiratory problems	18	8.5%
Upper/lower extremity complaint	13	6.2%
Abdominal pain	12	5.7%
Traumatic injury	11	5.2%
Convulsions/seizures	6	2.8%
Swallowed foreign body	4	1.9%
Body aches/myalgias	3	1.4%
Eye or ENT problem	3	1.4%
Other	14	6.6%
Total	211	100.0%

*ENT,* ear, nose, and throat

**Table 2:** Chief complaint by age, 6–18yrs.

Complaint	Total #	% of total
Abdominal pain	53	27.9%
Fever	39	20.5%
Traumatic injury	22	11.6%
Upper/lower extremity complaint	22	11.6%
Obstetrical complaint	11	5.8%
Vomiting and/or diarrhea	11	5.8%
Laceration	6	3.2%
Respiratory problem	5	2.6%
Convulsion/seizure	4	2.1%
Back pain	3	1.6%
Bites (human or animal)	3	1.6%
Other	11	5.8%
Total	190	100%

**Table 3:** Chief complaint by age, 19–59yrs.

Complaint	Total #	% of total
Abdominal pain	220	31.0%
Traumatic injury	88	12.4%
Back pain	58	8.2%
Fever	49	6.9%
Upper/lower extremity complaint	48	6.8%
Obstetrical complaint	46	6.5%
Vomiting and/or diarrhea	31	4.4%
Body aches/myalgias	20	2.8%
Chest pain	20	2.8%
Hemorrhage - unspecified	16	2.3%
Respiratory problems	13	1.8%
Genitourinary problem	13	1.8%
Eye or ENT problem	12	1.7%
Laceration	11	1.6%
Headache	11	1.6%
Bites (human or animal)	10	1.4%
Other	43	6.1%
Total	709	100.0%

*ENT,* ear, nose, and throat

**Table 4:** Chief complaint by age, 60+yrs.

Complaint	Total #	% of total
Abdominal pain	31	24.0%
Respiratory problems	13	10.1%
Traumatic injury	13	10.1%
Fever	10	7.8%
Vomiting and/or diarrhea	8	6.2%
Chest pain	7	5.4%
Back pain	5	3.9%
Neurological complaint	5	3.9%
Body aches/myalgias	4	3.1%
Fatigue/syncope	4	3.1%
Genitourinary problem	4	3.1%
Other	25	19.4%
Total	129	100.0%
